# The physiological functions of OPN5m

**DOI:** 10.1186/s40662-025-00467-9

**Published:** 2025-12-02

**Authors:** Jiali Ma, Sheikh Mizanur Rahaman, Yu Ohmura, Akihiro Yamanaka

**Affiliations:** 1 Beijing Institute for Brain Research, Chinese Academy of Medical Sciences & Peking Union Medical College, Beijing, 102206 China; 2 Academy for Advanced Interdisciplinary Studies, Peking University, Beijing, 100871 China; 3 Chinese Institute for Brain Research, Beijing (CIBR), Beijing, 102206 China; 4 Department of Neural Regulation, Nagoya University Graduate School of Medicine, Nagoya, 466-8550 Japan; 5 National Institute for Physiological Sciences, National Institutes of Natural Sciences, Aichi, 444-8585 Japan; 6 Division of Brain Sciences Institute for Advanced Medical Research, Keio University School of Medicine, Tokyo, 160-8582 Japan

**Keywords:** Non-visual photoreception, OPN5, G protein-coupled receptor, Retinal ganglion cells, Violet light, Hypothalamus, Thermoregulation, Myopia suppression, Circadian photoentrainment, Light-dependent signaling

## Abstract

Opsin 5 (OPN5), also known as neuropsin, is a violet/ultraviolet (UV) light-sensitive G protein-coupled receptor (GPCR) conserved across vertebrates. Most mammals possess a single OPN5 gene (*OPN5m*), whereas non-mammalian species also express OPN5L1 and OPN5L2 with distinct molecular properties. Mammalian OPN5 (OPN5m) functions as a non-visual photopigment, expressed in diverse extra-retinal tissues including the skin, testis, and brain. Recent studies reveal species-specific signaling: human OPN5m preferentially activates Gq-mediated Ca^2^⁺ signaling, mouse OPN5m couples with Gi to reduce cyclic adenosine monophosphate (cAMP), avian OPN5m engages either Gi or Gq depending on species and tissue, and amphibian/fish OPN5m homologs primarily signal through Gq pathways. These diverse signaling modes underlie a wide range of physiological functions, such as circadian photoentrainment, thermoregulation, vascular development, myopia suppression, corneal wound healing, seasonal reproduction in birds, and light-dependent hormone release in fish pituitary. As modern artificial lighting and indoor lifestyles limit violet light exposure, insufficient OPN5m activation may contribute to emerging health issues, particularly the global rise in myopia. This review provides an updated overview of the molecular diversity, expression patterns, signaling mechanisms, and physiological roles of OPN5m across species, and discusses its potential clinical relevance in the context of changing light environments.

## Background

For most species, photoreception, which is the sensing of environmental light conditions, provides crucial information necessary for health and survival. Photoreception involves an image-forming vision system as well as a variety of non-image-forming physiological processes including circadian rhythms, thermoregulation and mood [1–4].

Both visual and non-visual photoreception are achieved by opsins, which are universally conserved light-sensitive G protein-coupled receptors (GPCRs). Opsins are categorized into three groups based on function: photoisomerases, visual opsins, and non-visual opsins [5]. Photoisomerases, including retinal pigment epithelium (RPE)-retinal G-protein-coupled receptor (RGR) and peropsin, support the visual cycle by regenerating the chromophore. The physiological functions of human visual opsins—such as cone opsins (OPN1 family) and rhodopsin (OPN2)—are well characterized, whereas the molecular properties and physiological roles of non-visual opsins—including encephalopsin (OPN3), melanopsin (OPN4), and neuropsin (OPN5)— have yet to be fully characterized.

Among the non-visual opsins, OPN5 is the most recently identified. The *OPN5* gene was first cloned from the mouse and human genomes in 2003 [6]. Subsequent studies revealed that most mammals have a single *OPN5* gene (*OPN5m*), whereas non-mammalian vertebrates such as chickens, zebrafish, and *Xenopus* express *OPN5m* and two OPN5-like genes, *OPN5L1* and *OPN5L2* [7]. OPN5 shares the canonical seven-transmembrane structure of GPCRs and binds the vitamin A derivative retinal as its chromophore [6]. Mammalian OPN5 (OPN5m) and OPN5L2 are activated by 360 to 400 nm, a part of ultraviolet (UV) light, which is also termed violet light. OPN5m and OPN5L2 are sensitive to violet light and function in a light-dependent G protein signaling cascade [8]. Photoreception triggers isomerization of 11-*cis*-retinal to all-*trans*-retinal, initiating a transition from the resting to active state and activating downstream signaling via associated G proteins [6, 9, 10]. In contrast, OPN5L1 has different molecular characteristics: it binds all-*trans*-retinal in the dark-active state, and visible light exposure induces isomerization to 11-*cis*-retinal, and thus the molecule returns to its inactive conformation [11].

OPN5m in cultured cells is coupled to the Gi/o class of G proteins and contributes to a reduction in intracellular cyclic adenosine monophosphate (cAMP) levels [8]. However, recent evidence suggests that human OPN5m (hOPN5m) preferentially couples to Gq proteins, triggering the phospholipase C (PLC) pathway and mobilizing intracellular Ca^2^⁺ via the inositol 1,4,5-trisphosphate (IP₃) signaling cascade [12]. The complete signaling cascade and precise signaling mechanism of OPN5 are both not yet fully understood.

**Fig. 1:**
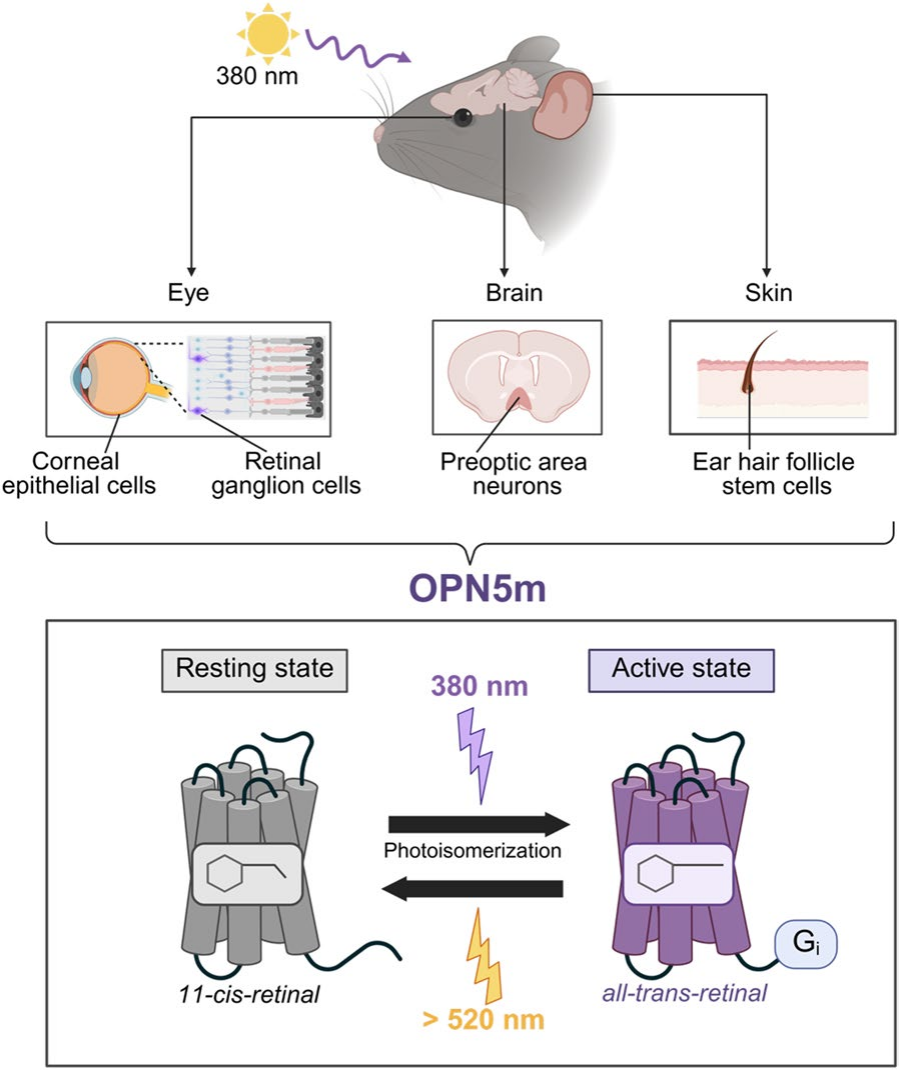
Expression pattern and molecular properties of mouse OPN5m. OPN5m is expressed in the eye, skin and brain of mice. Mouse OPN5m is a G-protein coupled receptor that selectively activates Gi proteins. OPN5m binds 11-*cis*-retinal in the resting state. Exposure to violet light induces a conformational change from 11*-cis-*retinal to all*-trans-*retinal and activation of coupled-Gi protein

OPN5m is considered to be a bistable opsin, since its active (all-*trans*-retinal-bound) and inactive (11-*cis*-retinal-bound) states are both thermally stable and interconvertible via light such that OPN5m does not require enzymatic chromophore regeneration. After activation by violet light illumination, subsequent exposure to orange light (> 520 nm) restores OPN5m to its resting state [8]. Notably, hOPN5m and mouse OPN5m (mOPN5m) cannot bind directly to all-*trans*-retinal due to a single amino acid substitution [13]. Retinal pigment epithelium-specific 65 kDa protein (RPE65), an essential enzyme in the retinal isomerization system, is expressed in the hypothalamic region near OPN5m-expressing cells [13]. This expression pattern suggests that OPN5m-expressing cells require an external supply of 11-*cis*-retinal to maintain photoreceptive function (Fig. 1).

In terms of spectral sensitivity, the maximum absorbance wavelength (λmax) of OPN5m falls in the violet to UV range. Both human and mOPN5m exhibit a λmax of ~ 380 nm [8], while chicken OPN5m (cOPN5m) has higher UV sensitivity with a λmax near 360 nm [10]. Meanwhile, other opsins in humans show peak absorption in the visible range: short-wavelength-sensitive cone opsins (OPN1sw) peak at ~ 420 nm, medium-wavelength (OPN1mw) at ~ 530 nm, and long-wavelength (OPN1lw) at ~ 560 nm [14]. Non-visual opsins like melanopsin (OPN4) peak at ~ 480 nm [15]. The spectral sensitivity of the non-visual opsin human encephalopsin (OPN3) has not been fully determined, but in other vertebrates like the zebrafish, chicken, and pufferfish, OPN3 was shown to absorb blue light at 465–470 nm [16, 17]. Given that the visible light spectrum for humans ranges from ~ 380 nm to 750 nm, and the human crystalline lens in the eye works as a filter to attenuate the transmission of short-wavelength light near 380 nm. The conserved expression of violet light-sensitive OPN5 suggests that it has a specialized role in non-visual photoreception, potentially functioning during early developmental stages before lens maturation.

OPN5 homologs have been identified in a broad range of species, including vertebrates, chordates, ambulacrarians, annelids, and brachiopods, indicating that the gene likely originated from a common bilaterian ancestor [18]. This evolutionary conservation suggests a fundamental role for OPN5 in photoreception and physiological regulation.

Notably, in mammals, OPN5m is predominantly expressed in extra-retinal tissues, such as the skin [19], reproductive organs [6], and deep brain regions including the hypothalamus [20] (Fig. 1). OPN5m is reported to mediate several non-visual light-dependent physiological functions such as photoentrainment in local circadian rhythm [21], thermogenesis [20], ocular vascular development [22], prevention of myopia [23], and wound healing in the cornea [24]. The spatial expression pattern, combined with its functional properties, supports the hypothesis that OPN5 primarily mediates non-image-forming photoreceptive functions, which are distinct from those of conventional visual pathways. In this review, we summarize the expression, intracellular signals, and physiological function of non-visual opsins in various species, with a focus on OPN5m.

## Main text

### Expression patterns of OPN5m

#### Mammals

OPN5m is reported to be expressed in the retina and cornea of mammals, from mice to primates [13, 20, 21, 24–26]. A recent study showed that OPN5m is selectively expressed in mammals’ retinal ganglion cells (RGCs) [25]. Other non-visual opsins, such as OPN3 and OPN4, are also expressed in RGCs [19, 26]. The functional roles of OPN4 in the retina, such as pupillary light reflex [27], are relatively well-examined. For example, a previous study using conventional knockout mice showed that OPN4 is involved in circadian photoentrainment [28]. In particular, glutamatergic inputs from OPN4-expressing cells in the retina, termed intrinsically photoreceptive retinal ganglion cells (ipRGCs), to the suprachiasmatic nucleus (SCN) are required for circadian photoentrainment [29]. Furthermore, ipRGCs project to different brain regions and are involved in distinct effects of light on learning and mood [30].

In contrast to OPN4, little is known about the role of OPN3 and OPN5 in the retina. Outside the retina, OPN3 appears to contribute to eye lens development [26, 31]. A recent study demonstrated that OPN5m expression facilitates wound healing in the cornea [24]. However, elucidating the function of OPN5m in the retina is challenging, given the very low expression levels in this tissue as well as the lack of commercially available, highly selective antibodies for OPN5 [21]. Some OPN5-specific antibodies have been described [8], but have not been widely validated [21]. Notably, much of the data concerning OPN5m expression is from studies using OPN5-cre recombinase mice crossed with Ai14 reporter mice, which express tdTomato, a red fluorescent protein, in a Cre-dependent manner.

These tdTomato-expressing cells were observed in the RGC and inner nuclear layer (INL) cells in adult mice [22, 25]. This subset of ganglion cells is thought to differ from the OPN4-expressing subset of ipRGC, suggesting that OPN5 has a different physiological role from that of OPN4 expressed in the retina. However, in this model, if OPN5 (Cre) is transiently expressed during the embryonic or fetal stage, the cells and their lineage cells will continue to be labeled with tdTomato even if OPN5 (Cre) is not subsequently expressed. In other words, some tdTomato-labeled cells may have previously expressed OPN5, suggesting that not all tdTomato-expressing cells necessarily express OPN5 at the time of observation. As such, whether OPN5 continues to be expressed in RGCs and INLs of mature mice, or what physiological roles these cells play, remain unclear.

Beyond the eye, several studies suggested that OPN5m is expressed in extraocular tissues in mammals, including the brain, skin, and testes [6, 32, 33], as other non-visual opsins [1]. In the brains of both young and adult mice, OPN5m expression is found exclusively in neurons of the hypothalamic preoptic area (POA) [13, 34]. However, during early development (before postnatal day 12) transient OPN5m expression may occur in the raphe pallidus. Nearly all OPN5-expressing neurons in the POA are glutamatergic and pituitary adenylate cyclase-activating peptide (PACAP)-positive (> 95%), and most also express mRNA of brain-derived neurotrophic factor (*Bdnf*) (> 80%). About half of these neurons express transient receptor potential cation channel, subfamily M, member2 (*TrpM2*), a heat-sensitive ion channel, whereas prostaglandin D2 synthase (*Ptgds*) expression is rare [20]. These patterns suggest that approximately half of OPN5 neurons play a role in thermoregulation. It should also be noted that OPN3 in adipocytes plays a role in thermogenesis [35, 36], implying a functional interaction between OPN3 and OPN5m.

In mammalian brains, OPN4 expression levels appear to be low or absent [37], but non-mammalian species like birds and reptiles do express OPN4 in the brain [38]. For OPN3, the expression is widely distributed across several regions of the brain [39]. Strong OPN3 expression is observed in the somatomotor/sensory areas, the ventral posteromedial nucleus of the thalamus, the dentate gyrus of the hippocampus, and the Purkinje layer of the cerebellum. OPN3 is expressed not only in neurons but also in astrocytes, especially in the dentate gyrus. The specific functional roles of OPN3 in these different brain regions are still unclear, but conventional knockout mice demonstrated that OPN3 is involved in eliciting the acoustic startle reflex [40].

In the skin, OPN5m is thought to be expressed mainly in the base of hair follicles of ear skin, where cells exhibit many markers typical of melanocyte progenitors. However, *OPN5* gene knockout in mice does not lead to changes in pigmentation, such as hyperpigmentation or albinism. *OPN5* expression has also been detected in dorsal and tail skin [33]. *OPN4* expression in mouse skin is relatively well established [41, 42], but *OPN4* expression in human skin remains controversial, with some studies detecting expression [43, 44], and other not [19, 45]. In contrast, most studies consistently showed that *OPN3* is expressed in human skin [19, 45, 46] where it might regulate blue light-induced melanogenesis, although its precise function in human skin is unclear [45, 46].

OPN5m is highly likely to be expressed in the testes of mammals, but the detailed localization of this expression is not known. One study reported OPN5m expression in sperm cells [47], but the specificity of the antibody used in this study has not been definitively confirmed. OPN3 and OPN4 expression is reported to be present in the testes of mammals [47, 48]. Some studies suggested that they may be involved in sperm movement, but again there is as yet no conclusive evidence due to issues with antibody selectivity.

#### Avian

In chicken, expression of the OPN5m homolog cOPN5m, in the retina begins during embryonic development, and, like OPN3, is expressed in RGCs and Müller glial cells. *OPN5* gene expression is detectable early in development (as early as embryonic day 7–10 in chicks) and continues through postnatal day 1 [7, 49, 50]. The expression likely continues over postnatal development based on a study reporting that *OPN5* gene expression could be detected in the eyes of adult border canaries [51].

OPN5m is also expressed in the brain and testes of birds. In quail brain, *OPN5* expression occurs in the paraventricular organ (PVO) within the mediobasal hypothalamus. cOPN5m in this brain region might mediate seasonal reproduction via direct activation by light, even without passing through the eyes, because exposure to UV or blue light promotes testicular growth in eye-patched quail [52]. Although the site of OPN5 expression is similar among different bird species, its function may differ. *OPN5* is also expressed in the mediobasal hypothalamus of border canaries, but transient knockdown using RNA interference increased photoinduced expression of thyrotropin-stimulating hormone β-subunit (TSHβ) in the hypothalamus, suggesting an inhibitory role for OPN5 in the timing of reproductive activation [51].

#### Amphibians and fish

Expression of XtOPN5m, the homolog of mammalian OPN5m in frogs, specifically in *Xenopus laevis*, begins during late embryonic development and continues into the larval stages, with a distinct retinal distribution and functional light sensitivity. OPN4, 6, and 8 are also expressed in the retina of frogs [53]. *OPN5* mRNA expression is first detected around developmental stages 37/38, coinciding with the initial activation of retinal circuits by light. At stage 41, *OPN5* is strongly expressed in cells of the outer region of the INL of the retina. Then, *OPN5* expression diminishes in the INL, while several *OPN5*-positive cells begin to appear in the ganglion cell layer [53]. *OPN5* is also expressed in the larval pineal complex, which is involved in skin pigmentation in response to environmental light [54].

A previous study comprehensively investigated OPN5m homologs in fish [55]. In ray-finned fishes like zebrafish, medaka (also known as Japanese rice fish), and spotted gar, OPN5 exists mainly as the paralogs OPN5m and OPN5m2. Unlike OPN5m, OPN5m2 exclusively binds 11-*cis*-retinal and not all-*trans*-retinal. OPN5m is predominantly expressed in the INL of the retina. When present (absent in medaka), OPN5m2 is mainly expressed at the outer edge of the INL. A small subset of OPN5-positive cells is also found in the ganglion cell layer. Beyond the retina, OPN5m is expressed in several regions of the brain, including the hypothalamus, which suggests that it plays a role in brain photoreception. In contrast, OPN5m2 expression in the brain of fish is more limited and species-specific; OPN5m2 is expressed in spotted gar, but not zebrafish. Table 1 summarizes OPN5 expression across species.

**Table 1:** OPN5 expression patterns across species

Organ	Mammals	Avians	Amphibians	Fish
Eyes	+	+	+	+
Brain	+	+	+	+
Skin	+	ND	ND	ND
Testes	+	ND	ND	ND

*ND* = not detected, no evidence that expression exists or expression has not been examined

### Intracellular OPN5 signals

**Fig. 2:**
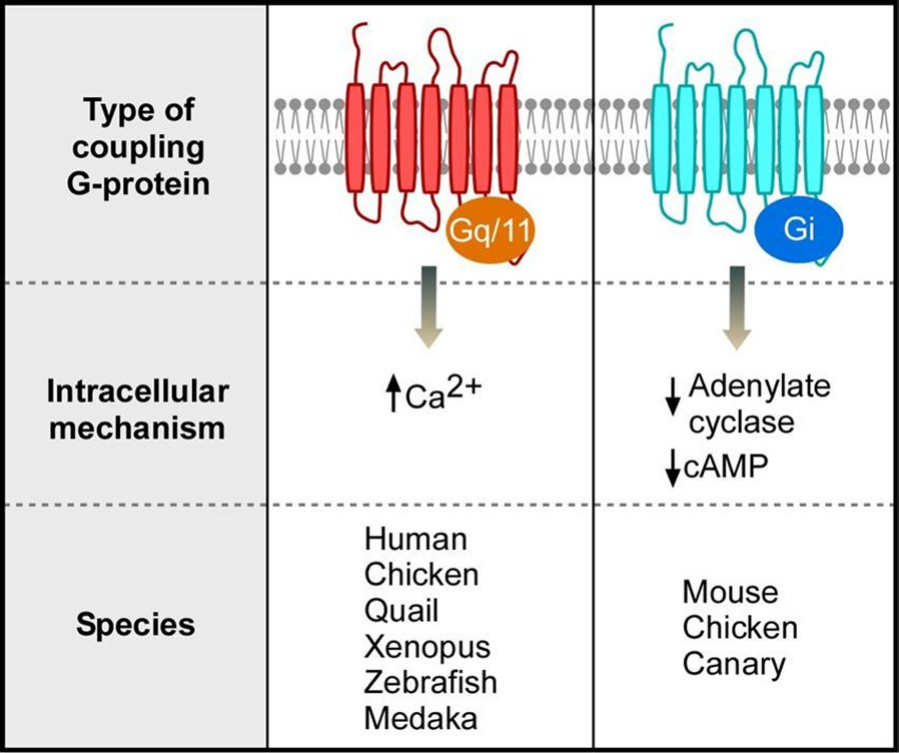
OPN5 signaling pathways across species

The intracellular signaling pathways involving OPN5 differ across species, but all rely on GPCR mechanisms. GPCR signals are mediated through coupled heterotrimeric G proteins that comprise α, β, and γ subunits (Fig. 2). The α subunit, which contains a GTP/GDP-binding domain, determines the signaling specificity of the receptor. The α subunits have four major classes: Gαi/o, Gαs, Gαq, and Gα12/13. Gαi/o inhibits adenylate cyclase to reduce intracellular cAMP levels, while Gαs activates adenylate cyclase to increase intracellular cAMP levels. Gαq stimulates PLC, which cleaves phosphatidylinositol 4,5-bisphosphate (PIP₂) into diacylglycerol (DAG) and IP₃. DAG activates protein kinase C (PKC), whereas IP₃ binds to its receptors on the endoplasmic reticulum to trigger calcium (Ca^2^⁺) release into the cytoplasm [56, 57]. OPN5m and OPN5L2 function as violet light-sensitive GPCRs and are widely expressed in mammals, whereas OPN5L1 is a visible light-sensitive opsin that contributes to non-visual photoreception and light-dependent physiological processes in birds, amphibians, and fish [58]. The following sections provide a species-specific overview of the intracellular signaling pathways mediated by OPN5.

#### Mammals

hOPN5m functions as a light-sensitive GPCR that responds primarily to UV light and plays a role in modulating intracellular signaling pathways. Early studies in HEK293 and Neuro2A cells revealed that brief UV stimulation of hOPN5m led to a transient release of Ca^2^⁺ from intracellular stores and a reduction in intracellular cAMP, both with and without exogenous 11-*cis*-retinal. UV light exposure also triggered MAPK phosphorylation, suggesting that hOPN5m modulates multiple intracellular pathways that mediate Ca^2^⁺ signaling, cAMP levels, and MAPK activation [59]. Subsequent mechanistic studies using genetic knockout models and pharmacological tools clarified the G protein coupling specificity of hOPN5m [57]. These studies demonstrated that UV-induced Ca^2^⁺ transients depend on G_q/11_ and not G_i_ proteins as evidenced by the persistence of light-induced Ca^2^⁺ responses in G_i_ knockout cells but not in G_q/11_ knockout cells. Additionally, Gβγ subunits are known to interact with G protein-coupled inwardly rectifying K^+^ channel (GIRK) channels. GIRK channel opening induces hyperpolarization of the membrane potential by allowing K^+^ ions to flow from the intracellular space to the extracellular space. GIRK channel assays that are typically used to monitor G_i_ activity showed that UV light actually inhibited GIRK currents in hOPN5m-expressing cells. This effect remained in the presence of G_i_ inhibition but was abolished by G_q_ inhibition, further indicating that hOPN5m does not couple with Gi proteins expressed in the HEK293 culture cells but instead selectively signals through the G_q_ pathway, particularly via G_q14_ [12, 57].

On the other hand, mOPN5m is a UV and violet-light-sensitive GPCR that is known to selectively couple with G_i_-type G proteins. In OPN5m-expressing HEK293S cells, exposure to UV light substantially reduces intracellular cAMP levels, consistent with G_i_ protein activation, and this reduction in turn inhibits adenylate cyclase activity. This light-dependent suppression of cAMP confirms the role of OPN5m as a G_i_-coupled receptor in mammalian cells and supports its function in regulating non-visual phototransduction pathways [8]. Beyond its signaling mechanism, mOPN5m plays a critical physiological role in postnatal vascular development in the eye. Nguyen and colleagues [22] discovered a light-dependent pathway initiated by OPN5m in RGCs that controls hyaloid vessel regression, a necessary developmental process. Activation of OPN5m by near-UV light lowers dopamine levels in the vitreous via vesicular GABA transporter (VGAT) and dopamine transporter (DAT) phosphorylation, and this lowering of dopamine in turn activates dopamine D2 receptor (DRD2). This signaling cascade suppresses vascular endothelial growth factor 2 (VEGFR2) activity in hyaloid vascular endothelial cells, thereby inhibiting abnormal vessel growth.

#### Avian

Avian OPN5 exhibits light-dependent, G protein-coupled signaling that varies by species and context, with functional implications in both neuronal excitability and reproductive regulation. Chicken OPN5 (cOPN5), a homolog of OPN5m (cOPN5m), is a light-sensitive GPCR that couples primarily with Gi-type G proteins in the presence of 11-*cis*-retinal, as shown in chick tissues. cOPN5m localizes in the RGC layer, pineal gland, and PVO—regions that are associated with dopaminergic and serotonergic signaling. A GTPγS-based biochemical assay demonstrated that cOPN5m activates Gi signaling, suggesting its role in non-visual photoreception, in which light-dependent Gi pathways may regulate neurochemical processes and circadian functions [10]. In contrast, when cOPN5m is heterologously expressed in mammalian cells (e.g., HEK293T cells and mouse astrocytes), it can be repurposed to activate Gq-type signaling upon blue light stimulation. This activation leads to rapid intracellular Ca^2^⁺ release and PKC activation, enabling precise optogenetic control of cellular activity at subcellular resolution. Furthermore, optogenetic manipulation using cOPN5m has been successfully applied to modulate neuronal activity and control behavior in a circuit-specific manner [60].

Electrophysiological recordings from Xenopus oocytes expressing quail OPN5m revealed that short-wavelength (420 nm) light stimulation triggers a Ca^2^⁺-activated Cl⁻ current, mediated by chloride channels that respond to intracellular Ca^2^⁺ elevation. This Ca^2^⁺ influx results in membrane depolarization, consistent with Gq-type protein activation and subsequent PLC-Ca^2^⁺ signaling, which plays a role in regulating seasonal reproduction [52]. Similarly, whole-cell patch clamp recordings of OPN5m-expressing PVO neurons confirmed that short-wavelength light rapidly induces depolarization [61]. These findings demonstrate that quail OPN5m can couple to Gq pathways, leading to excitation in response to light.

In contrast, studies in canaries revealed a different physiological function for OPN5. In canaries, OPN5m (a homolog of cOPN5m) is expressed in the mediobasal hypothalamus, testis, and eye, where it plays a role in regulating seasonal reproduction. During the photoinducible period, RNAi-mediated knockdown of OPN5m expression resulted in a significant increase in thyroid-stimulating hormone (TSH)β mRNA, a marker of reproductive activation. This finding indicates that OPN5m normally inhibits TSHβ expression, thereby acting as a negative regulator of photoperiod-induced reproductive responses. The inhibitory effect aligns with Gi/o protein signaling, suggesting that, in canaries, OPN5m couples with Gi proteins to suppress reproductive hormone signaling [51].

#### Amphibians and fish

OPN5 is expressed in various non-mammalian vertebrates, including amphibians like *Xenopus tropicalis* and teleost fishlike zebrafish and medaka. In these species, the OPN5 homologs XtOPN5m (*X. tropicalis*), DrOPN5m and DrOPN5m2 (zebrafish), and OlOPN5m (medaka) have been identified [57]. Although the intracellular signaling pathways of non-mammalian OPN5 variants are not fully characterized, recent studies suggest that they couple primarily with Gq-type G proteins. Notably, light stimulation of these opsins is associated with increased intracellular Ca^2^⁺ levels, with enhanced responses observed in the presence of Gα14, indicating a conserved Gq-mediated signaling mechanism across vertebrate OPN5m homologs [57].

A recent study uncovered a novel light-responsive endocrine function of pituitary melanotrophs in medaka. Among the 34 opsins examined, *OPN5m* alone was expressed in the posterior pituitary, the region where melanotrophs localize. Activation of OlOPN5m by short-wavelength UV light (365–460 nm) induces a rapid intracellular Ca^2^⁺ increase, triggering the exocytosis of melanocyte-stimulating hormone (MSH). This hormone release promotes melanogenesis in the skin by upregulating tyrosinase and associated proteins, suggesting a UV-dependent photoprotective mechanism. These findings establish a non-visual, opsin-mediated autonomous hormonal response in the pituitary and broaden the functional scope of opsins beyond the central nervous system to affect deeper endocrine organs [62].

Taken together, OPN5 is a UV-sensitive GPCR that is conserved across mammals, birds, amphibians, and fish and mediates non-visual photoreception through species-specific signaling pathways. While OPN5 often couples to Gi/o proteins to reduce cAMP and PKA activity, some species have Gq coupling that triggers PLC activation and intracellular Ca^2^⁺ release. In humans, OPN5 signals mediated via Gq elevate Ca^2^⁺ and activate MAPK, whereas in mice OPN5 couples to Gi to lower cAMP and regulate eye vascular development. Avian OPN5 shows both Gi and Gq coupling depending on the context and species. Gi functions in chicken and canary tissues, whereas Gq functions in quail and heterologous systems. In amphibians and fish, OPN5m homologs primarily couple to Gq, especially Gα14, supporting a conserved Ca^2^⁺ signaling mechanism in lower vertebrates. Figure 2 provides a brief overview of OPN5 interactions with different G-protein subtypes, and Table 2 presents a broad summary of OPN5 intracellular pathways and their functions across various species.

**Table 2:** Comparative overview of OPN5 signaling and biological functions

Species	OPN5 variant	Primary G protein coupling	Intracellular mechanism	Physiological functions	Reference
Human	hOPN5m	Gq (Gq14)	↑ Ca^2^⁺ (Gq-dependent), ↓ cAMP (initially suggested, later excluded), ↑ MAPK	Non-visual photoreception, photoentrainment, control myopia	12, 49, 56, 58
Mouse	mOPN5m	Gi	↓ cAMP (via adenylate cyclase inhibition) ↑ cAMP	Circadian rhythm, Thermoregulation, Control myopia	8, 20, 21, 23, 62
Chicken	cOPN5m	Gi (native) Gq (heterologous)	↓ cAMP in native tissue (Gi), ↑ Ca^2^⁺ in HEK cells (Gq)	Non-visual photoreception	10
Quail	cOPN5m	Gq	↑ Ca^2^⁺ → Ca^2^⁺-activated Cl⁻ current → depolarization	Deep brain photoreceptor, seasonal reproduction	51
Canary	cOPN5m	Gi	↓ TSHβ expression (Gi)	Seasonal reproduction	50
Xenopus	XtOPN5m	Gq	↑ Ca^2^⁺	Skin pigmentation, deep brain photoreception	57, 63, 64
Zebrafish	DrOPN5m/ DrOPN5m2	Gq (Gq14 > others)	↑ Ca^2^⁺	Retinal and pineal photoreception	57
Medaka	OlOPN5m	Gq (Gq14 > others)	↑ Ca^2^⁺, ↑ MSH	Skin melanogenesis	61

*OPN5* = opsin5; *OPN5m* = mammalian OPN5; *hOPN5m* = human OPN5m; *mOPN5m* = mouse OPN5m; *cOPN5m* = chicken OPN5m; *XtOPN5m* = xenopus OPN5m; *DrOPN5m* = zebrafish OPN5m; *OIOPN5m* = medaka OPN5m; *cAMP* = cyclic adenosine monophosphate; *MAPK* = mitogen-activated protein kinase; *HEK* = human embryonic kidney; *TSHβ* = thyrotropin-stimulating hormone β-subunit; *MSH* = melanocyte-stimulating hormone

### Physiological function of OPN5m

As mentioned above, OPN5 is expressed in many organs and tissues. In mammals, OPN5m is mainly expressed in the skin, retina, and brain, and functions in sensing short-wavelength light, including violet light. In the skin, OPN5m is expressed in ear (pinna) and vibrissal pad skin. In hair bulbs, OPN5m-expressing cells include hair follicle stem cells and melanocyte progenitor cells. OPN5m senses UV light and stimulates the synthesis of melanin (melanogenesis), which effectively absorbs UV [63]. The synthesized melanin is transported to keratinocytes to protect the skin from harmful UV exposure. An ex vivo photoentrainment experiment showed that OPN5m also functions in the photoentrainment of skin circadian clocks [21]. This function is consistent with OPN5m expression in ear skin and vibrissal pad skin that are frequently exposed to ambient light. However, whether photoentrainment that affects skin circadian clocks contributes to photoentrainment at the behavioral level to affect the internal rhythm of the SCN is as yet unclear.

Meanwhile, Ota et al. generated OPN5-null mice to show that OPN5m indeed plays a role in the circadian photoentrainment at the behavioral level [64]. These mice exhibited impaired photoentrainment and phase shifting to UVA light. However, triple-knockout mice lacking all known functional circadian photoreceptors (i.e., rods, cones, and melanopsin) did not entrain to UVA-light/dark cycles, despite the presence of OPN5m [64]. This finding indicates that expression of OPN5m alone is not sufficient for photoentrainment at the behavioral level and that functional interactions with other photoreceptors are required to fully regulate circadian rhythm. Further studies are needed to clarify both the expression localization and detailed role of OPN5m in photoentrainment of the circadian clock.

Nguyen et al. reported that OPN5-dependent retinal light responses regulate vascular development in the postnatal eye [22]. They showed that hyaloid vessels in OPN5-null mice show precocious regression mediated through OPN5 in RGCs that in turn enhance the activity of the inner retinal DAT and suppresses vitreal dopamine. The action of dopamine on hyaloid vascular endothelial cells suppresses VEGFR2 activity and promotes hyaloid vessel regression.

Jiang et al. reported that OPN5 has a role in suppressing lens-induced myopia by sensing violet light in mice [23]. Therefore, exposure to short-wavelength visible violet light may have an important role in preventing myopia progression by activating OPN5 in the retina. The authors showed that lens-induced myopia was ameliorated by exposure to violet light in mice and that the effect of violet light was attenuated in mice with conditional OPN5 knockout in the eye achieved by crossing Chx10-cre mice that express Cre in the eye with OPN5-floxed mice. The molecular mechanism by which exposure to violet light suppresses myopia and how expression of OPN5 in certain cell types in the eye is involved in this process is unclear, but may involve OPN5m-mediated increases in early growth response protein 1 (EGR-1) expression in the retina, which is observed in mice and chickens exposed to violet light [50, 65]. Further research is needed to elucidate the details of these mechanisms.

The hypothalamus in the brain also has robust OPN5m expression. Many OPN5m-expressing cells are located in the POA that is located in the deepest area of the brain, which, in mammals, may be largely inaccessible to violet light that must first penetrate multiple tissues including the hair, skin, skull, and brain. However, Zhang et al. showed that OPN5m can sense violet light at the POA and contribute to regulating body temperature [20]. They showed that OPN5m-expressing cells in the POA are mainly glutamatergic neurons. Chemogenetic activation or inhibition of these neurons significantly decreased and increased body temperature, respectively. Additionally, ex vivo experiments using neurons expressing a FRET-based cAMP sensor suggest that violet light illumination directly increases intracellular cAMP levels. Together, these findings suggest that OPN5m is sensing violet light at the POA and regulates metabolism and heat generation for efficient regulation of body temperature. Whether sufficient light intensity from natural light reaches the POA after passage through other tissues, and whether OPN5m senses this light and contributes to physiological temperature regulation is currently unclear and further studies are needed to define the molecular mechanisms and roles of OPN5m at the neural circuit level.

In *Xenopus laevis*, XtOPN5m is expressed in the retina and skin, as well as the pineal complex and diencephalon of the brain [66, 67]. In situ hybridization revealed that in the *X. laevis* retina, XtOPN5m mRNA is distributed across multiple retinal cell types, including bipolar (~ 70% to 75%), amacrine (~ 10%), and retinal ganglion (~ 20%) cells [53]. Light illumination induced c-Fos expression in these cells, suggesting that XtOPN5m is contributing to light sensation in the *X. laevis* retina. In the brain, XtOPN5m and/or cryptochrome 1-expressing cells are located in a small region of the caudal diencephalon. These cells sense short wavelength UV light (400 nm) and contribute to the induction of regular bouts of rhythmic swimming activity [67].

A recent study showed that exposure of medaka fish to short-wavelength light induces an increase in intracellular calcium and release of melanocyte-stimulating hormone [62]. In medaka fish, OlOPN5m is expressed in the pituitary [55]. OlOPN5m knockout attenuated melanogenesis by reducing tyrosinase expression in the skin, suggesting that OlOPN5m is mainly involved in this response by directly sensing violet light at the pituitary. Medaka fish have relatively transparent bodies, and thus violet light could penetrate into deep areas of the brain, including the pituitary. This OPN5m-mediated effect on tyrosinase expression represents a physiologically reasonable response of medaka fish to violet light and UV light exposure that produces melanin to protect cells from UV light.

In quail, cOPN5m is expressed in neurons of the deep areas of the brain that contact the cerebrospinal fluid (CSF). This interaction contributes to sensing light at 419 nm and regulating the release of TSH for seasonal reproductive activity [52].

### Conclusion

OPN5m is expressed in various parts of the body in multiple species, where it detects violet light and regulates physical responses that are critical for maintaining homeostasis. As research progresses in various animal species in the future, our understanding of the physiological role of OPN5m will continue to advance.

Light in the environment of modern societies has undergone dramatic changes over the past decades. For example, humans now spend large amounts of time indoors and have less exposure to natural light. Moreover, some window glass can block short-wavelength visible light, including violet light. Artificial lighting like LED lights used in interiors lacks short-wavelength visible light, including violet light. Hence, our exposure to short-wavelength visible light has been greatly reduced. The rapid changes in external ambient light associated with modern society could lead to insufficient activation of OPN5m that may in turn induce various biological abnormalities. For myopia in particular, children who spend more time outdoors are reported to have a lower incidence of myopia [68]. Recent findings that violet light suppresses myopia progression through OPN5m activation are remarkably consistent with these facts [23].

On the other hand, we are now exposed to blue light emitted from LCD monitors and other devices during both the day and night. As a result, various issues have arisen due to the loss of its functions originally performed by non-visual opsins or the excessive and inappropriate timing of stimuli.

Thoroughly elucidating the physiological role of OPN5m and the mechanisms of its involvement in pathophysiology should facilitate the development of methods and therapeutics to prevent and treat issues associated with OPN5m.

## Data Availability

Not applicable.

## Abbreviations

GPCR: G protein-coupled receptor
OPN5m: Mammalian OPN5
UV: Ultraviolet
cAMP: Cyclic adenosine monophosphate
hOPN5m: Human OPN5m
PLC: Phospholipase C
RPE65: Retinal pigment epithelium-specific 65 kDa protein
OPN1sw: Short-wavelength-sensitive cone opsins
OPN1mw: Medium-wavelength-sensitive cone opsins
RGCs: Retinal ganglion cells
ipRGCs: Intrinsically photoreceptive retinal ganglion cells
SCN: Suprachiasmatic nucleus
INL: Inner nuclear layer
POA: Preoptic area
PACAP: Pituitary adenylate cyclase-activating peptide
Bdnf: Brain-derived neurotrophic factor
TrpM2: Transient receptor potential cation channel, subfamily M, member2
Ptgds: Prostaglandin D2 synthase
PVO: Paraventricular organ
TSHβ: Thyrotropin-stimulating hormone β-subunit
PIP₂: Phosphatidylinositol 4,5-bisphosphate
DAG: Diacylglycerol
IP₃: Inositol 1,4,5-trisphosphate
PKC: Protein kinase C
GIRK: G protein-coupled inwardly rectifying K+ channel
mOPN5m: Mouse OPN5m
VGAT: Vesicular GABA transporter
DAT: Dopamine transporter
DRD2: Dopamine D2 receptor
VEGFR2: Vascular endothelial growth factor 2
cOPN5m: Chicken OPN5m
EGR-1: Early growth response protein 1
